# Animal Models of Hepatocellular Carcinoma: The Role of Immune System and Tumor Microenvironment

**DOI:** 10.3390/cancers11101487

**Published:** 2019-10-02

**Authors:** Zuzana Macek Jilkova, Keerthi Kurma, Thomas Decaens

**Affiliations:** 1 Université Grenoble Alpes, CS 10217, 38043 Grenoble, France; Keerthi.Kurma143@gmail.com; 2 Institute for Advanced Biosciences, Research Center UGA/Inserm U 1209/CNRS 5309, 38043 Grenoble, France; 3 Clinique Universitaire d’Hépato-gastroentérologie, Pôle Digidune, CHU Grenoble Alpes, CS 10217, 38043 Grenoble, France

**Keywords:** animal model, hepatocellular carcinoma, cancer, immune system, tumor microenvironment

## Abstract

Hepatocellular carcinoma (HCC) is the most common type of liver cancer in adults and has one of the highest mortality rates of solid cancers. Ninety percent of HCCs are associated with liver fibrosis or cirrhosis developed from chronic liver injuries. The immune system of the liver contributes to the severity of the necrotic-inflammatory tissue damage, the establishment of fibrosis and cirrhosis, and the disease progression towards HCC. Immunotherapies have emerged as an exciting strategy for HCC treatment, but their effect is limited, and an extensive translation research is urgently needed to enhance anti-tumor efficacy and clinical success. Establishing HCC animal models that are analogous to human disease settings, i.e., mimicking the tumor microenvironment of HCC, is extremely challenging. Hence, this review discusses different animal models of HCC by summarizing their advantages and their limits with a specific focus on the role of the immune system and tumor microenvironment.

## 1. Introduction

**Figure 1:**
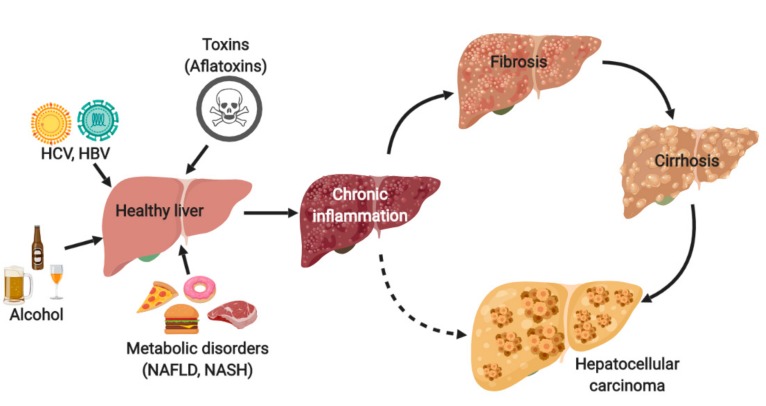
Risk factors and the process leading to the development of hepatocellular carcinoma (HCC). Hepatitis C virus, HCV; hepatitis B virus, HBV; non-alcoholic fatty liver disease, NAFLD; non-alcoholic steatohepatitis, NASH.

Hepatocellular carcinoma (HCC) is the most common type of liver cancer in adults and has one of the highest mortality rates of solid cancers. The incidence of HCC has been rising over the past 20 years and will soon surpass one million annual cases worldwide [1]. Viral chronic infection with hepatitis B virus (HBV) or hepatitis C virus (HCV), aflatoxin-contaminated foodstuffs, chronic alcohol consumption, and metabolic disorders are the major causes of chronic liver inflammation which leads to fibrosis or cirrhosis, or both, and finally to HCC development (see Figure 1). Even though the distribution of these risk factors is highly variable, depending on the geographic region or ethnic group, 90% of HCC cases are always developed in the background of chronic inflammation and fibrosis/cirrhosis. The immune system of the liver plays a crucial role and inherently contributes to the severity of the necrotic-inflammatory damage, the establishment of liver fibrosis, and disease progression towards HCC [2,3].

Nowadays, less than 30% of patients with HCC are diagnosed at the early stages, when potentially curative treatments (i.e., resection, liver transplantation, and local ablation) are applicable [4]. On the other hand, the majority of patients who are diagnosed at an advanced stage have limited treatment options and, thus, the prognosis of HCC remains very poor. Sorafenib emerged in 2007 as the first effective systemic treatment of HCC for patients with advanced HCC or those progressing from locoregional therapies. However, the objective response rate to sorafenib is exceedingly low (2%). More recently, several new drugs have shown positive clinical results in first- or second-line setting therapies, as reviewed elsewhere [5]. In addition, immunotherapies, mainly the agents targeting the PD-1/PD-L1 pathway and its combinations with other treatments, have a good chance to significantly improve HCC therapeutic strategies in the future [6]. Despite this progress, new treatments of HCC with a better efficacy remain urgently needed.

Unfortunately, the process of anti-HCC drug discovery and development seems to be very challenging and inefficient as reflected by the high attrition rate of drugs that enter preclinical testing but fail to gain FDA approval [7]. One of the underlying causes is the low predictive value of animal models of HCC that are used before in-human clinical trials are launched. In this review, we have described the different animal models of HCC available, summarizing their advantages and their limits, with a specific focus on their capacity to mirror the human immune system and tumor microenvironment.

## 2. Animal Models of HCC

Animal experimentation has played a crucial role in cancer research throughout history. As in other areas of cancer research, rodent animal models, especially mice, have become increasingly important in the field of HCC, mainly due to their short lifespan and breeding capacity [8]. However, it is important to mention that every HCC animal model is artificial in some way. Establishing potent animal models that mimic human HCC settings is particularly challenging, due to complex etiology, tumor heterogeneity, and the importance of both chronic inflammation and fibrotic background of human HCC.

HCC animal models can be categorized as follows: (i) chemically induced models, (ii) genetically engineered models, (iii) syngeneic models, (iv) xenograft models including patient-derived xenograft models, and (v) humanized models. The majority of these models can be combined with specific diets to generate NASH-associated HCC as recently reviewed elsewhere [9,10].

**Figure 2:**
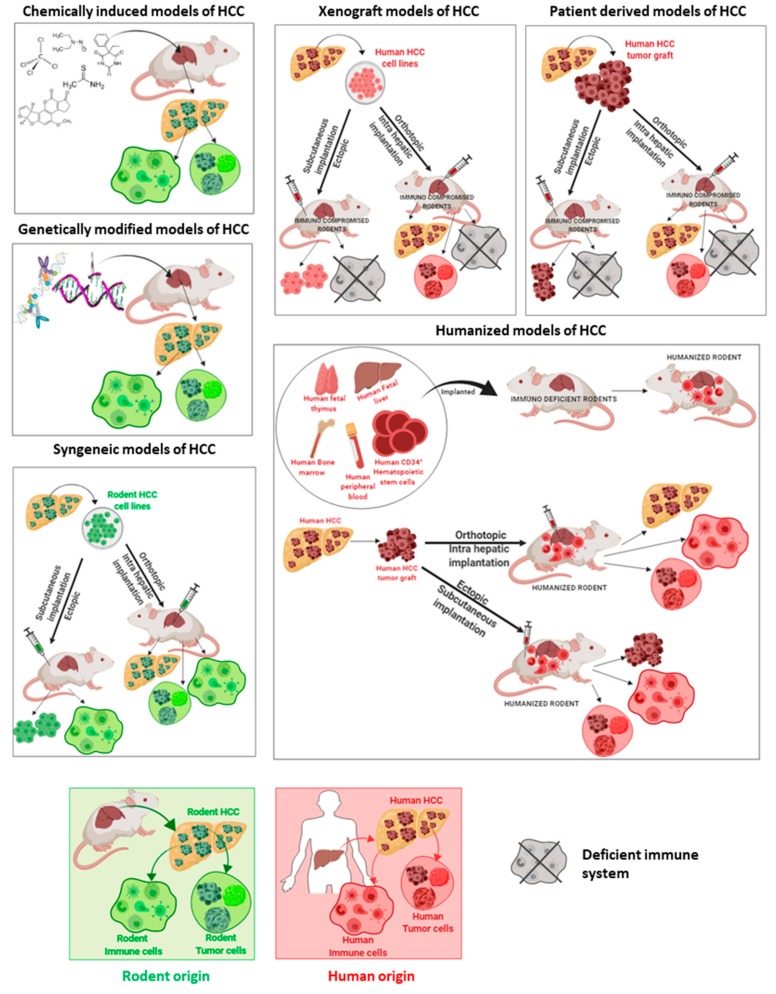
Rodent models of HCC and the origin of immune cells and tumor cells. Rodent HCC, rodent immune cells, and rodent tumor cells (green color); human HCC, human immune cells, and human tumor cells (red color).

The origin of immune cells and tumor cells differ between animal models of HCC, as shown in Figure 2, which can represent the main limitation, depending on the type of research that is planned.

Additionally, the knowledge of the pros and the cons of each HCC animal model is essential for obtaining results that are meaningful for the HCC field and for clinical translation, see Table 1.

### 2.1. Chemically Induced Models

The first chemically induced model of HCC was developed by the Japanese researcher, Riojun Kinosita, who performed a series of experiments (1932–1937) demonstrating that 4-dimethyl-aminoazobenzene strongly induces liver cancer in rats [11]. Since then, chemically induced animal models have been widely used. These models have provided a physiologically relevant tissue microenvironment and immune modifications related to HCC development and progression. Thus, chemically induced animal models are reliable in revealing underlying mechanisms of carcinogenesis, such as genetic, environmental, and immunological factors that influence cancer susceptibility in the human population [12]. The important disadvantage of chemically induced models is the long time taken for tumor induction and the undefined genetic background of the tumor. Furthermore, this is one of the reasons why the use of these models has diminished in the last decades. However, it is precisely the prolonged time that facilitates the development of the chronic inflammatory environmental characteristics of human HCC, including fibrosis, leading to tumor development and progression in the future.

Several chemical compounds are able to induce carcinogenesis after acute, short, or long-term exposure, depending on the chemical structure, concentration, animal susceptibility, etc. Based on their activity and specific pathogenic mechanism, chemical carcinogenic compounds are categorized as either genotoxic carcinogens or non-genotoxic hepatocarcinogens [13].

#### 2.1.1. Genotoxic Carcinogen Induced Models

Genotoxic or direct-acting carcinogens (such as diethylnitrosamine (DEN) or aflatoxins) interact directly with DNA through the formation of covalent bonds, resulting in DNA-carcinogen complexes (DNA adducts). If chronically injected, DEN induces chronic inflammation and then fibrosis. Thus, genotoxic carcinogens are frequently used to induce liver fibrosis and HCC in rodents.

Specifically, after the administration to an animal, DEN is metabolized in the centrilobular hepatocytes, followed by reactions that cause DNA damage [14], which is associated with oxidative stress. These principle metabolizing pathways induced by DEN in rodent models are similar to that of humans [15]. Therefore, the application of DEN has become highly attractive for studies that are aimed at understanding the pathogenetic alterations underlying the formation of liver cancer.

However, HCC development by DEN depends on specific characteristics, such as species, dosage of administration, or the age and sex of the rodents. All these factors impact, among others, the tumor microenvironment and immune status.

A classical mouse model of DEN-induced HCC uses a single injection of a low dose of DEN as an initiator. However, such a model does not develop the features of liver fibrosis, which is crucial to mimic tumor microenvironment of HCC in humans. Therefore, a single injection of DEN is usually accompanied by repeated dosing of a pro-fibrogenic agent CCl_4_ [16]. A classical rat model of DEN-induced HCC is based on chronic exposures to DEN in developing fibrosis to cirrhosis, followed by HCC after 14–20 weeks [17,18,19]. Furthermore, this accurately recapitulates the scenario of human HCC. Additionally, the functional genomics showed that the gene expression patterns of DEN-induced HCCs were extremely similar to those of the poorer survival group of human HCCs [20]. Recently, the sequencing and analysis of gene copy number changes, similar to morphological findings (abundant inclusion bodies, fatty change), showed that tumors of the DEN model match with human tumor samples of alcohol-induced HCC [21].

In addition, the incidence of DEN-induced HCC development is gender-dependent, with a high prevalence in male rodents, which is similar to humans. In fact, several studies have demonstrated that female mice and rats are largely resistant to DEN-driven hepatocarcinogenesis. This phenomenon is related to estrogen-mediated inhibition of IL-6 production by Kupffer cells in females [22], which clearly sustains the relevance of the immune system in this model.

Indeed, the DEN induced model was used to study the role of immune response and tumor microenvironment in the process of hepatocarcinogenesis. The combination of DEN and CCl_4_ was employed in a landmark study in which the contribution of the toll-like receptor 4 (TLR4) signaling on HCC promotion was investigated [23]. Furthermore, the DEN-induced mouse model was used to deeply characterize anti-tumor adaptive immune responses and the role of T and B cells in controlling tumor formation and progression [24]. Recently, the DEN-induced cirrhotic rat model of HCC served as a relevant model for the detailed study of the modulations of tumor microenvironment and immune system by the AKT inhibitor [19]. Additionally, the DEN-induced HCC rat model was used to recapitulate portal hypertension and gut permeability, which are two key players in the pathogenesis of HCC [25].

In contrast with the DEN-induced HCC models, aflatoxin-induced animal models are rarely used. In fact, even though aflatoxin B_1_ exposure in food is one of the major risk factors for HCC development in human population [26], aflatoxin-induced genetic alterations of mice models differ from aflatoxin-related human HCC, as susceptibility to HCC development in animals is highly variable, and the development of the tumor takes from several months to a year [27].

#### 2.1.2. Non-Genotoxic Carcinogen Induced Models

Non-genotoxic carcinogens, such as carbon tetrachloride (CCl_4_), thioacetamide (TAA), and phenobarbital and peroxisome proliferators have no direct interaction with DNA. These treatments cause hepatic damage by disrupting cellular structures and altering the kinetics of either cell proliferation or of processes that increase the risk of genetic error, which leads to inflammation-associated events, liver fibrosis, and the increased incidence of HCC. Moreover, non-genotoxic carcinogens are usually combined with DEN to develop reproducible mice models of HCC in the context of fibrosis. Furthermore, the CCl_4_ model is the best characterized with respect to immunological changes associated with the development of fibrosis [28] and it is wildly used to develop fibrosis in other animal models of HCC.

### 2.2. Genetically Modified Models

The development of transgenic and gene targeting technologies in the past decades facilitated the generation of genetically modified models (GMMs) to study tumor biology. GMMs have become powerful research animal models as they allow insight into the involvement of specific proteins and signaling pathways in the generation of HCC [29]. In this scenario, the main advantage of GMMs is the knowledge of the initiating mutation, which is particularly important for the testing of molecularly targeted anti-HCC therapies. In addition, HCC spontaneously develops in these models in a coevolving liver microenvironment and the immune system is intact. 

However, one important disadvantage of GMMs is the frequent absence of fibrosis/cirrhosis and often the low tumor mutation burden, as the tumors develop from limited genetic alterations. In contrary, human HCC usually develops in the background of fibrosis and it is known to be heterogeneous with an extensive landscape of altered genes and pathways [30,31,32]. Thus, although genetically engineered models of HCC have brought a precise step toward recapitulating the human HCC, the resultant spontaneous tumors without chronic inflammation and fibrosis do not recapitulate the stepwise progression of human HCC. Additionally, it is important to mention that the majority of GMMs are time consuming and expensive, as extensive infrastructures are required to achieve sufficient population, especially for preclinical studies. Today, with the advent of clustered regularly interspaced short palindromic repeats (CRISPR)/Cas technology, it is expected that some of these drawbacks could be improved, as editing is relatively fast, which could enhance the value of GMMs. However, CRISPR/Cas technology should be used on the background of liver fibrosis or cirrhosis to correctly mirror the human HCC.

The GMMs of HCC are models that overexpress oncogenes, growth factors, or express viral genes. These approaches need to be quite often combined to obtain shorter latency and increased tumor induction. For instance, β-catenin pathway is thought to be most frequently activated in human HCCs [32] but the second hit from an additional mutation is required to generate HCC in β-catenin transgenic animals. These genetic models of β-catenin–induced hepatocellular transformation were used to characterize the specific inflammatory response and to demonstrate the inflammation as a key player in β-catenin–induced liver tumorigenesis [33]. Additionally, transgenic model targeting only c-Myc proto-oncogene develops cancer in 60–70% of animals [21,34]. The tumors of this model display high heterogeneity and well mimic alcohol-induced HCCs, based on genomic changes [21]. The coexpression of c-Myc with Tgfα in transgenic mouse model leads to a tremendous acceleration of neoplastic development and progressive hepatocyte proliferation [21] on the background of chronic oxidative stress and persistent disruption of immune microenvironment in all animals [34,35]. Furthermore, the gene expression patterns of the c-Myc/Tgfα transgenic mouse model were shown to be extremely similar to those of the poor survival group of human HCCs [20]. This model may help to study the importance of the cross-talk between the tumor microenvironment and immune effector cells in the process of aggressive HCC. Similarly, double transgenic mice (c-Myc OVA tg+) c-Myc-OVA-tTALAP is a model of multifocal and rapidly progressing aggressive HCC. It was used to test combinations of three immunostimulatory monoclonal antibodies targeting OX40, PD-L1, CD137, and adoptive therapy with specific tumor antigen-activated T cells showing a clear synergy between the triple combination of mAbs and adoptive transfer of anti-tumor-specific cells [36]. Recently, a novel Akt1/N-Ras-induced HCC mouse model was generated to elucidate crosstalk between tumor-associated antigen-specific T cells and stromal cells, and the underlying mechanisms governing immunosuppression in the HCC tumor microenvironment [37].

The transgenic model with aberrant expression of the cytokine lymphotoxin (AlbLTαβ model) was crucial to discover a lymphotoxin-driven pathway to HCC and more generally, the inflammation-induced hepatocarcinogenesis [38]. Additionally, tumors from AlbLTαβ transgenic mice revealed a high overlap with NASH-HCC, based on genomic changes [21].

Different types of GMMs were developed to study HBV and HCV infections but these manipulations did not always lead to HCC. In fact, the models of HBV-associated hepatocarcinogenesis usually show long latency and low tumor induction [39]. Still, these models provide the opportunity to study the role of the immune system on spontaneous HCC development in the context of HBV infections. For instance, HBsAg-transgenic (HBs-tg) mice were recently used to demonstrate the importance of HBV-related adaptive immune tolerance, showing the importance of an immune checkpoint, TIGIT, whose blocking or deficiency led to fibrosis and HCC [40]. Concerning HCV-associated hepatocarcinogenesis, the FL-N/35 mouse model, expressing the full HCV genome, is certainly a relevant transgenic mouse model, especially when combined with CCl_4_ for investigating fibrosis and HCC development [41,42].

### 2.3. Syngeneic Models

Syngeneic animal models have been used for interventional studies in HCC since the last decade. In these models, HCC cells from the same species are injected to the immunocompetent animals, usually directly to the liver tissue that can be simultaneously stimulated to develop fibrosis in order to mimic a true HCC microenvironment. Thus, these models are particularly valuable, as they give a possibility to investigate all key players of the immune system and tumor microenvironment, including vasculature, stroma and surrounding lymph system [43]. However, the disadvantage of syngeneic models can be their limited similarity to human HCC as the commonly used cell lines are often driven by mutations that do not mimic those found in human HCC.

It is important to mention that syngeneic orthotopic models of HCC often result in the development of metastases [44], which makes these models particularly interesting for studying the suppression of antitumor immune response during metastasis promotion. Furthermore, due to the functional system of tumor–immune surveillance, syngeneic rat models with a spontaneous tumor regression were used to show the involvement of particular cytokines in an effective anti-tumor immune response [45]. Similarly, the syngeneic mouse orthotopic model of HCC was used to study immunosuppressive properties of hepatic stellate cells and their contribution to the development of HCC [46]. In addition, today, syngeneic models combined with CCl_4_ or TAA treatment are well-established models of fibrosis-associated HCC. Recently, these models were used to study the importance of the fibrotic microenvironment and myeloid-derived suppressor cells for the sensitivity of HCC to immune checkpoint therapies [47] or to test new combination therapies targeting tumor microenvironment [48].

### 2.4. Xenograft Models

In classical xenograft models, HCC is established either by the inoculation of human tumor cell lines or the direct implantation of a fragment of human solid tumors subcutaneously or into the liver of the immunodeficient animal.

Cell-line ectopic xenograft models, where human HCC cells are implanted mostly subcutaneously, have been extensively used in the HCC field for decades. The relative ease and the rapidity by which these models are prepared makes them a compelling preclinical model to screen cytotoxic drugs. However, the results obtained with these models have often inadequately predicted human clinical outcomes, as reviewed elsewhere [49,50]. One of the given reasons is the need to grow tumors in immunodeficient mice, which are not reflective of the dynamic process of tumor–immune surveillance. Thus, the functional immune system is missing in these models. Second, these models do not take the liver microenvironment into account. This leads to the model where the tumor microenvironment is extremely artificial with the complete absence of the surrounding fibrotic tissue. Another common concern is that HCC cells change in culture over time and do not always stably recapitulate the phenotypes or genotypes of human HCC. Nowadays, when targeting tumor microenvironment and immune system becomes a compelling way to tackle HCC, researchers are turning to other models as cell-line ectopic xenograft models have evident limitations for such investigation.

Orthotopic xenograft models reflect the tumor microenvironment, especially the influence of liver vascularization but have many of the same limitations as classical ectopic xenograft models. Mainly, the use of the immunodeficient host does not allow one to study the immunomodulatory effects of drugs in these models.

To better preserve the natural disease state observed in HCC patients, some groups xenograft freshly resected pieces of HCC into immunodeficient animals. This procedure, known as patient-derived xenograft (PDX), preserves histopathologic, transcriptomic, and genomic characteristics of the original HCC and can often well recapitulate the chemotherapeutic drug response. Thus, the PDX models have demonstrated an important utility for the evaluation of personalized precision medicine [51]. The most common is subcutaneous implantation, but, of course, the orthotopic PDX models of HCC better replicate tumor microenvironment and, thus, are more physiologically relevant. However, the PDX models of fibrosis-associated HCC are not yet available. The general limitations of the use of the PDX models are the long lag period necessary for engraftment and passaging and the high costs of PDX development, maintenance and testing. Nevertheless, the real “Achilles heel” of the PDX models is the lack of a functional immune system, which is common for all xenograft models.

The incorporation of PDX models in HCC research brings substantial improvements and many interesting liver cancer PDX models had been generated and used for preclinical testing of anticancer drugs [51,52,53]. Recently, Blumer et al. established several PDX models from human HCC biopsies, showing that PDX tumors can retain characteristics of the original HCC biopsies over six generations of retransplantation [54].

### 2.5. Humanized Animals: Future Models of HCC?

During the last decades, anti-HCC drug development has focused on targets and partially de-emphasized the importance of immune system. Today, the tumor-immune system interface is required to test the majority of treatments. Furthermore, an immune system of the host is required especially for testing of new immunotherapeutic options. In general, the main limitation of animal models is that they do not accurately recapitulate a functional human immune system. Therefore, efforts have been made to create rodents with a human immune system (a “humanized rodent”) for immunotherapeutic efficacy testing.

The ultimate goal of humanization is to generate animals expressing human immune cells that mimic realistic tumor-immune system interactions, which are mainly capable of mounting anticancer immune responses for specific immunotherapeutic interventions. The first successful engraftments of human hematopoietic stem cells [55] or human peripheral blood mononuclear cells [56] were established in the late 1980s. Today, due to improvements in the development of immune-deficient rodents, several types of humanized model are routinely employed in cancer research such as: (i) engraftment of human peripheral blood lymphocytes to severe combined (SC) immunodeficient rodents; (ii) engraftment of human CD34^+^ hematopoietic stem cells acquired from multiple sources, such as bone marrow, fetal liver, or umbilical cord to SC immunodeficient rodents, or (iii) engraftments of 16–22-week gestation fragments of human fetal liver and human fetal thymus to sub renal capsular space of SC immunodeficient rodents. Although the current progress and advances in the humanization of animals are remarkable, the main limitations include the high cost and technical difficulties. Moreover, in the current generation of humanized rodents, certain human immune subpopulations are incompletely reconstructed. For instance, human myeloid cells are usually under-represented or have functional defects [57].

Even though humanized rodents represent one of the most attractive preclinical models for the screening of therapies targeting tumor-immune system interface, they are not yet well established in the HCC field. Thus, more experience with these models in HCC research are urgently needed to improve HCC immunotherapy research.

Recently, Zhao et al. developed a new PDX humanized mouse model to study human-specific tumor microenvironment and immunotherapy. In fact, they xenografted HCC subcutaneously into a mouse model of type I human leucocyte antigen that matched the human immune system in NOD-SC immunodeficient rodents Il2rg−/− (NSG) mice and studied the immune responses as well as the efficacy of immune checkpoint inhibitors [58]. Taking into consideration the advantage of humanized PDX models, the orthotopic humanized PDX models will mainly present in the future an extremely attractive option for studying how a functional human immune system reacts with the tumor in order to reproduce the complexity and specificity in humans HCC of novel immunotherapeutic targets.

## 3. Conclusions

We come from a period when ectopic xenograft growing in immune-deficient animals were considered sufficient for anti-HCC drug screening. Today, the boom of therapies targeting the immune system and tumor microenvironment highlight the importance of the host, background of chronic inflammation, and fibrosis. Hopefully, the development and the use of animal models with respect to these principles will improve our capacity to effectively develop and screen novel anti-HCC drugs.

## Figures and Tables

**Table 1:** Summary of the pros and the cons of animal models of HCC.

Animal Models of HCC	Pros	Cons	Origin of Immune System
Chemically induced	Functional tumor-immune system interface Chronic inflammation Presence of fibrosis/cirrhosis	Long-time of tumor induction Undefined genetic background of the tumor	Animal immune system
Genetically engineered	Specific gene mutation Functional tumor-immune system interface	Low tumor mutational burden Development of fibrosis/cirrhosis needs to be stimulated	Animal immune system
Syngeneic	Functional tumor-immune system interface Metastasis formation	Limited similarity to human HCC Development of fibrosis/cirrhosis needs to be stimulated	Animal immune system
Xenograft	Low cost and rapid Predictable tumor growth	Unsuitable for studying tumor-immune system interface Absence of fibrosis/cirrhosis	Deficient immune system
Humanized	Recapitulate the tumor-immune system interface of human origin Highly relevant for the testing of immunotherapies	Incomplete reconstitution of the human immune system The high cost and technical difficulties Absence of fibrosis/cirrhosis in the models of today	Human immune system
